# Spontaneous Dissections of Bilateral Internal Carotid and Vertebral Arteries due to Intractable Vomiting

**DOI:** 10.1155/2022/8156047

**Published:** 2022-04-11

**Authors:** Gift Echefu, Rameela Mahat, Raju Vatsavai, Steven Zuckerman

**Affiliations:** ^1^Baton Rouge General Medical Centre, Internal Medicine Residency, Baton Rouge, LA, USA; ^2^Hospital Medicine Group, Baton Rouge General Medical Center, Baton Rouge, LA, USA; ^3^Baton Rouge Clinic Neurology, Baton Rouge, LA, USA

## Abstract

In young adults, spontaneous craniocervical arterial dissections (sCAD), which involve the major arteries of the head and neck, are associated with an increased risk of stroke. sCAD occurs in the absence of major trauma as seen in traumatic craniocervical artery dissection. It may affect unilateral or bilateral carotid or vertebral arteries. Cases of spontaneous bilateral carotid and vertebral artery dissections occurring simultaneously are extremely rare. We present a case of a 49-year-old female with no history of arteriopathy who presented with aphasia and right upper extremity weakness and was found to have dissections in bilateral extracranial and intracranial carotid arteries, as well as the bilateral vertebral arteries. She had symptomatic improvement with antithrombotic therapy and aggressive outpatient rehabilitation.

## 1. Introduction

Craniocervical artery dissection (CAD) may result directly from trauma such as in motor vehicle accidents or spontaneously in the absence of major trauma. It may also occur spontaneously following minor traumas including neck manipulation, recent respiratory tract infection, violent coughing, or may have no discernable preceding event [1]. Spontaneous carotid artery stenosis (sCAD) may involve the carotid or vertebral arteries either unilaterally or bilaterally, and in rare instances, all craniocervical vessels may be involved. Spontaneous internal carotid artery dissection is twice as common as vertebral artery dissection. VA is more vulnerable to dissection at its entry point into the transverse foramen of 6^th^ cervical vertebrae, atlantoaxial, and atlanto-occipital junctions owing to the high mobility of the vessels at these areas [2].

The true incidence of sCAD is uncertain but has been estimated at 2.5-3 per 100,000 people in carotid artery dissection and 1-1.5 per 100,000 people in vertebral artery dissection [3, 4]. It is a rare cause of stroke with most cases occurring in young adults less than 50 years [5]. Overall, carotid artery dissection, including traumatic and spontaneous cases, accounts for 2% of all cerebrovascular accidents (CVAs) [3, 6]. Patients may present with the classic triad of unilateral face, neck, or head pain; partial Horner's syndrome was followed by cerebral or retinal ischemia days later [1]. We present a case of bilateral sCAD presenting with aphasia and right upper extremity numbness in a patient with violent vomiting.

## 2. Case Presentation

A 49-year-old woman presented to our hospital with sudden onset difficulty with speaking. The previous day, she had complained of left sided facial and neck pain as well as right upper extremity numbness to her family. Her past medical history was significant for major depressive disorder, migraine, insomnia, and chronic lower back pain. She had a viral illness 4 days prior to presentation manifesting with upper respiratory symptoms and multiple episodes of vomiting and diarrhea. She had no recent history of trauma or neck manipulation. On physical examination, she was febrile (101.2 °F) and tachycardic, her neurological exam revealed an expressive aphasia, but was able to write on paper. Motor exam showed 5/5 power in all extremities, with normal gait. The rest of the physical examination was normal.

**Figure 1:**
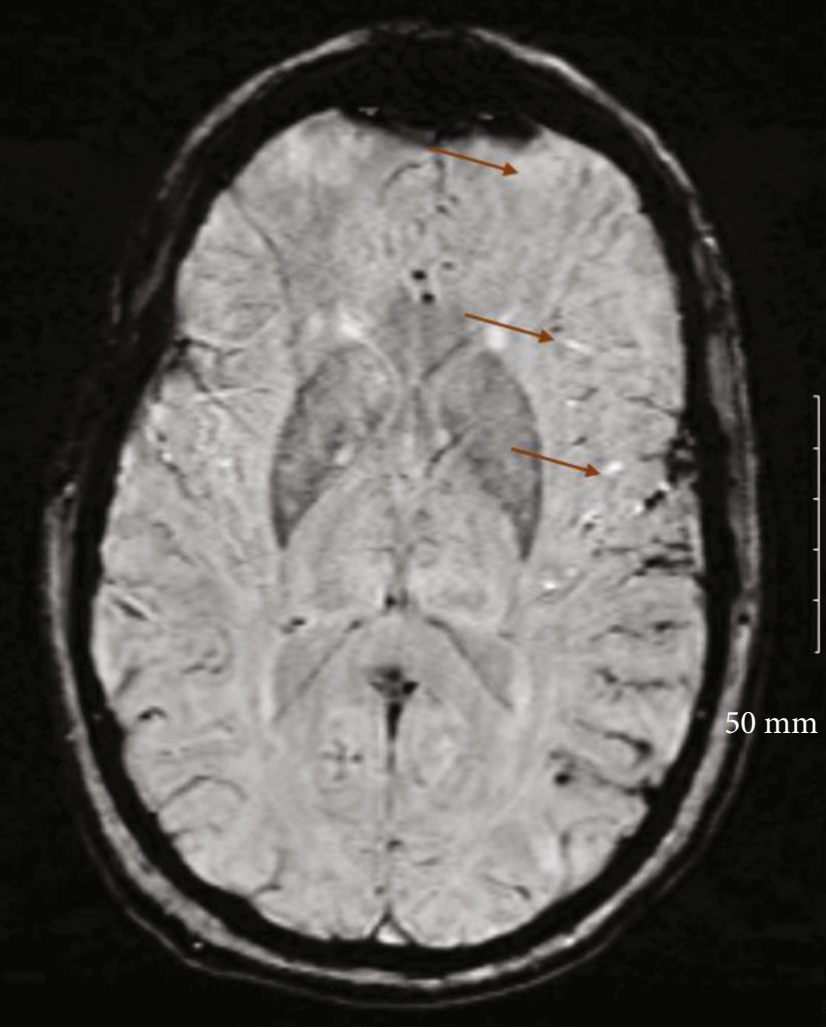
Susceptibility weighted magnetic resonance imaging showing the multifocal left frontal and parietal cortical infarction.

**Figure 2:**
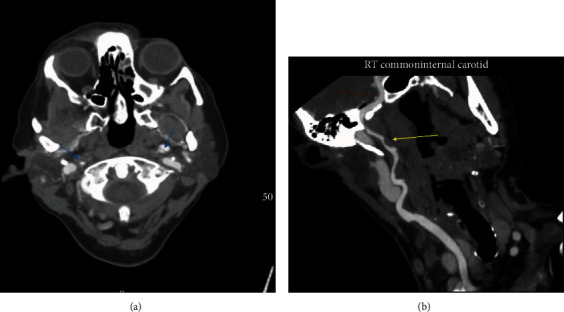
(a) Computed tomography angiography imaging showing the right and left common carotid artery dissection (blue arrows). (b) Computed tomography angiography imaging showing the right common (yellow arrow) and internal (red arrow) carotid artery dissections.

She was admitted to the telemetry unit. Workup to investigate stroke risk factors and mechanism including coagulopathy, toxicology screening for drugs, glycosylated hemoglobin (A1c), lipid panel, thyroid function test, and vasculitis antibodies were all normal. Bilateral carotid doppler ultrasound showed increased velocities in the internal carotid arteries bilaterally, but no stenosis. Magnetic resonance imaging of the head was obtained which revealed multiple bi-hemispheric infarcts, predominantly affecting the left frontal, parietal lobes, and right internal capsule (Figure 1). CT angiogram of the head (Figures 2(a) and 2(b)) obtained revealed bilateral common, internal carotid artery dissections, and pseudoaneurysms with high grade stenosis and near total occlusion of the distal internal carotids bilaterally.

**Figure 3:**
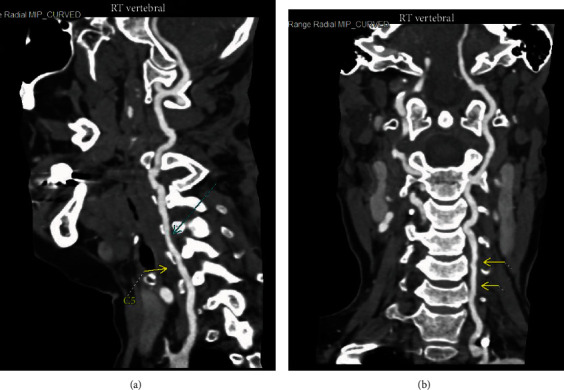
(a) Computed tomography angiography imaging showing the right vertebral dissection (cyan arrow). (b) Computed tomography angiography imaging showing the left vertebral artery dissection (yellow arrows).

Neurology and vascular surgery were consulted, and anticoagulation with apixaban was recommended. Her speech and right upper extremity numbness continued to improve with physical therapy, and she was discharged on day 4 with an outpatient physical therapy arranged. However, she returned the day following discharge with intractable emesis, worsening speech impairment, and new right upper extremity weakness. Repeat CTA head and neck revealed progression of intimal dissection with slight interval progression of stenosis and irregularity of the ascending cervical internal carotid arteries bilaterally. There were new foci of grade 1 vascular injury involving the V2 segment of the bilateral vertebral arteries near the levels of C4-C5 and C5-C6 not present on the imaging during previous admission (Figures 3(a) and 3(b)). Due to worsening neurological deficits and new vertebral dissection, she was transferred to a comprehensive stroke center for evaluation for possible endovascular intervention. There, apixaban was continued with close observation in the intensive care unit. The neurologists and vascular surgeons at the stroke center recommended continuing apixaban. At discharge from the stroke center, apixaban was discontinued, and she was initiated on aspirin and clopidogrel. Our patient had improvement in her neurological deficits with outpatient rehabilitation, dual antiplatelet therapy (DAPT), and no new symptoms following discharge. She was seen in the clinic at 2 and 9 months with follow-up imaging revealing improvement in the dissections and pseudoaneurysm.

## 3. Discussion

Spontaneous cervical artery dissection is rare overall across all age groups but is not an uncommon cause of ischemic stroke in people below 50 years [5]. Stroke mechanism in sCAD includes local thrombus formation due to intimal injury and release of thrombogenic factors resulting in artery-to-artery embolization and ischemic infarction. Another stroke mechanism is hemodynamic compromise from intramural hematoma causing luminal stenosis and downstream infarction. Arterial dissection results in the narrowing of the lumen, and as the dissection progresses, the already thin adventitia becomes weaker and out pouches resulting in pseudoaneurysm or arteriovenous fistulas with potential for bleeding [7].

Spontaneous bilateral internal carotid artery dissection is rare, and simultaneous occurrence with vertebral artery dissection is even rarer. In a population of 740 patients with arterial dissection, Arnold et al. [8] found 11 cases of triple sCAD (1.6% of all sCAD) and a single case of quadruple sCAD (0.1%). Arterial wall disease was suggested as a predisposing factor in the etiology of sCAD in patients with triple and quadruple sCAD, and these patients were more likely to present with headaches due to associated vascular dysfunction [8]. The authors also reported higher proportion of sCAD among men with hypertension. Women were younger and more likely to present with multiple dissections, which they suggested could be due to genetic factors, hormones, oral contraceptives, and pregnancies [8].

The proposed risk for spontaneous carotid artery dissection is that the structural defect in the arterial extracellular matrix may predispose to dissection in susceptible vessels, due to a combination of environmental and genetic factors [9]. Still, most cases of spontaneous carotid artery dissections are considered idiopathic. Certain conditions such as vasculitis or connective tissue disorders including Ehlers-Danlos syndrome, Marfan syndrome, and fibromuscular dysplasia, especially in women, confer an increased risk of sCAD due to intrinsic vascular structure abnormality [10, 11]. Therefore, these should be evaluated during workup in patients with sCAD to establish etiology.

Isolated cases of sCAD usually, unilateral, in individuals with normal carotids have been reported after nontraumatic events like cough, vomiting, chiropractic manipulation, yoga, sneezing, receiving anesthesia, following resuscitative measures, and noncontact sports [12]. The mechanism is thought to be due to neck hyperextension or rotation with resultant injury to the artery from mechanical stretching during these abrupt neck movements.

Acute infection has also been reported as an independent risk factor in the pathogenesis of sCAD. Guillon et al. [13] described a prospective study of 47 patients with sCAD and 52 with ischemic stroke from other causes and found an association between recent infection and SCAD compared with other types of cerebral ischemic events [13]. sCAD in this setting tended to involve multiple vessels. Several authors linking inflammation to sCAD have proposed several mechanisms, including indirect inflammation and host reponse, cytokine and protease induced extracellular matrix degradation of the vascular wall, prothrombotic, oxidative and autoimmune mechanisms [1, 13].

Our patient had no diagnosis of structural vascular defect or fibromuscular dysplasia and had no personal or family history of spontaneous artery dissection or notable change in lifestyle prior to the event. It is likely that in our patient, the mechanical factor due to violent vomiting and indirect inflammation from viral infection may have rendered the vessels vulnerable to dissection.

Neurological deficits from sCAD usually develop hours to days after initial symptoms and may be transient, making diagnosis difficult [14]. The most common presenting symptoms are unilateral head, facial, or neck pain, which occurs in 57 to 90% of cases and may precede the neurological deficits [15–17]. The patients may also present with visual deficits from retinal infarction or partial Horner's syndrome (miosis and ptosis only). Cranial nerve palsies, especially hypoglossal nerve and partial Horner's syndrome, tend to occur due to local compression of the structures [15, 16, 18].

The diagnosis of sCAD is confirmed using imaging studies that visualize the vessels and direction of blood flow. CTA or MRI of the brain with MRA can help establish the diagnosis [19]. Transcranial doppler ultrasound can also be used, but visualization may be limited, as dissection can only be observed when the vascular lumen is reduced to at least 50%. MRI should be obtained with MRA as this increases its sensitivity to 99% [7]. In our case, initial MRI without MRA revealed bilateral embolic infarcts which led to workup for embolic stroke of undetermined source (ESUS). Subsequent CTA was able to localize the dissected segments.

Expansion and recurrence of dissection impact morbidity and mortality and should be carefully monitored. Early recurrence has been reported to occur at a higher rate within the first month, sometimes within days, declining to around 1% per year after the first year [20]. Recurrent dissections were reportedly more common in younger patients and tended not to involve the same vessel [21]. Our patient had bilateral internal carotid artery dissection during the initial evaluation, but repeat imaging after 4 days revealed an extension of the intimal tear and a new vertebral artery dissection. The true mortality rate associated with sCAD remains difficult to estimate, but has been reported to be low with no reported gender difference [8].

Antithrombotic therapy is the management of choice in sCAD, often with good outcomes. The CADISS (Cervical Artery Dissection in Stroke Study) trial found no significant differences in outcomes between antiplatelet therapy versus anticoagulation for stroke patients with sCAD [22]. This is similar to the outcome of a more recent investigation by the TREAT-CAD trial investigators [23]. Endovascular interventional therapy, in the form of angioplasty and stent placement, is reserved for patients with contraindication to antithrombotic therapy; progressive, profound ischemic symptoms; expanding pseudoaneurysm; or hemodynamic instability [9].

## 4. Conclusion

Spontaneous cervical artery dissection should be considered a potential mechanism of stroke in young patients without traditional risk factors for ischemic stroke. Prompt imaging with magnetic resonance imaging with angiography or CT angiogram should be considered to prevent delay in diagnosis. The recurrence rate is high within the first few days to a month of event and should be suspected in any case of worsening or new neurological deficits.
